# Aging Remodels Mitochondrial Architecture Across the Male Reproductive Axis

**DOI:** 10.21203/rs.3.rs-9802113/v1

**Published:** 2026-05-26

**Authors:** Suzanna Navarro, Claude F. Albritton, Estevao Scudese, Victoria Ryen Baskerville, Mohd Khan, Calixto Pablo Hernandez Perez, Max Kushner, Andrea G. Marshall, Edgar Garza Lopez, Berwin Singh Swami Vetha, Prasanna Venkhatesh, Suraj Thapliyal, Prasanna Katti, Tadeu Sixel Santana, Jenny Schafer, Bret Mobley, Julia Berry, Debra D. Murray, Annet Kirabo, Celestine Wanjalla, Melanie McReynolds, Joyonna Gamble-George, Antentor Hinton

**Affiliations:** Department of Molecular Physiology and Biophysics, Vanderbilt University, Nashville, TN, USA; Department of Medicine, Division of Clinical Pharmacology, Vanderbilt University Medical Center, Nashville, TN, USA; Laboratory of Biosciences of Human Motricity (LABIMH), Federal University of the State of Rio de Janeiro (UNIRIO), Rio de Janeiro, Brazil; Sport Sciences and Exercise Laboratory (LaCEE), Catholic University of Petrópolis (UCP), Brazil; Department of Biochemistry and Molecular Biology, The Huck Institute of the Life Sciences, Pennsylvania State University, State College, PA 16801, USA; 3Department of Biomedical Sciences, School of Graduate Studies, Meharry Medical College, Nashville, TN, USA; Department of Molecular Physiology and Biophysics, Vanderbilt University, Nashville, TN, USA; Department of Cell and Developmental Biology, Vanderbilt University, Nashville, TN, USA; Department of Molecular Physiology and Biophysics, Vanderbilt University, Nashville, TN, USA; Department of Biomedical Sciences, School of Graduate Studies, Meharry Medical College, Nashville, TN, USA; Department of Pharmacology and Toxicology; Department of Foundational Sciences, East Carolina University, Greenville, NC, USA; Indian Institute of Science Education and Research (IISER) Tirupati; Indian Institute of Science Education and Research (IISER) Tirupati; Indian Institute of Science Education and Research (IISER) Tirupati; Laboratory of Biosciences of Human Motricity (LABIMH), Federal University of the State of Rio de Janeiro (UNIRIO), Rio de Janeiro, Brazil; Sport Sciences and Exercise Laboratory (LaCEE), Catholic University of Petrópolis (UCP), Brazil; Department of Cell and Developmental Biology, Vanderbilt University, Nashville, TN, USA; Department of Pathology, Vanderbilt University, Nashville, TN, USA; Department of Pathology, Vanderbilt University, Nashville, TN, USA; Department of Molecular and Human Genetics, Baylor College of Medicine, Houston, TX 77030, USA; Department of Molecular Physiology and Biophysics, Vanderbilt University, Nashville, TN, USA; Division of Infectious Diseases, Vanderbilt University Medical Center, Nashville, TN 37232, USA; Department of Biochemistry and Molecular Biology, The Huck Institute of the Life Sciences, Pennsylvania State University, State College, PA 16801, USA; Department of Computer Science, Whiting School of Engineering, Johns Hopkins University, Baltimore, MD, USA; Department of Molecular Physiology and Biophysics, Vanderbilt University, Nashville, TN, USA

**Keywords:** MICOS, mitochondria, male infertility, multiomics, microscopy, pharmacology

## Abstract

Male infertility is recognized as a complex condition associated with aging, yet its association with mitochondrial structural remodeling is poorly understood. Here, we integrated a multi-model approach to investigate how aging and MICOS complex dysfunction influence male reproductive health. Using the *All of Us* Research Program, we analyzed 121,064 male participants and performed age stratified PheWAS, LabWAS, and GWAS analyses comparing young (20 to 40 years) and older (≥55 years) cohorts. Male infertility was associated with distinct age dependent clinical phenotypes, laboratory alterations, and candidate loci linked to mitochondrial dysfunction. We examined semen samples using confocal microscopy and transmission electron microscopy upon inhibition of MICOS, which resulted in impaired sperm motility dynamics while increasing oxidative stress. We also used *Drosophila melanogaster* expressing mitochondrial green fluorescent protein in testes. Older flies displayed significant alterations in spermatid mitochondrial architecture, indicating disruption of mitochondrial network organization through aging. Complementary serial block face scanning electron microscopy and three-dimensional reconstructions of mouse testes revealed extensive age associated mitochondrial remodeling across residual, loose, and germ cell populations. Together, these findings identify mitochondrial MICOS complex integrity as a central regulator of age-associated male infertility across species, highlighting it as potential therapeutic target for age-related reproductive decline.

## INTRODUCTION

It is widely known that male reproductive health functions, most notably fertility, steeply decline with age. This is in part due to diminished sperm count, which is brought on by many age-related factors. One such factor is the decrease in the number of Sertoli cells and thus germ cells since Sertoli cells nourish germ cells as they are developing into sperm cells. Leydig cell count also decreases, and as Leydig cells produce testosterone, there is a resulting decrease in testosterone levels. Furthermore, since testosterone helps produce sperm, a decrease in testosterone reduces sperm count. There is also increased secretion of the gonadotropins, namely follicle-stimulating hormone (FSH) and luteinizing hormone (LH), which is associated with various types of testicular disorders and failures. These decreased levels of testosterone and increased levels of gonadotropins ultimately result in decreased mean testicular volume with age, which is a result of decreased numbers of testicular cells and has the added effect of keeping sperm count low as there is less space for the sperm. A diminished sperm quality is also observed, manifested in decreased sperm motility and increased DNA fragmentation, the latter effectively inducing increased genetic mutations in sperm.^1^

Broadly, the testis plays important roles in spermatogenesis and testosterone production, and it exhibits age-related decreases in steroidogenesis, sperm count, and sperm quality.^2^ Morphological changes associated with aging in the testis and sperm have also been documented. However, most parameters indicative of abnormal morphology other than decreases in volume have not been well studied^1^ and the mitochondrial quantifications that have been conducted have been in 2D.^3^ Therefore, we employed 3D and 2D imaging methods to provide a holistic view of the effects of aging on mitochondrial and sperm morphology and sperm motility. We quantified mitochondrial structural changes associated with aging using Serial Block Face-Scanning Electron Microscopy (SBF-SEM) and additional morphological and motility shifts assessed by transmission electron microscopy (TEM) and confocal microscopy. We hypothesized that aging causes structural remodeling of mitochondrial architecture in the testis and sperm, leading to mitochondrial dysfunction and decreased overall reproductive health.

At the organelle level, mitochondrial aging is known to be associated with fragmented mitochondria as this fragmentation is the result of the increased mitochondrial fission that accompanies aging.^4^ In particular, a host of dynamin-related guanosine triphosphatases (GTPases) regulate the fusion and fission cycle of mitochondria. For example, optic atrophy-1 (OPA1), an inner mitochondrial membrane protein that promotes mitochondrial fusion,^5^ is lost with age,^6,7^ thereby stimulating fission. Additional consequences of age-related changes in protein concentrations include loss of OPA1, leading to reduced mitochondrial calcium uptake and retention capacity,^8^ and an increase in mitochondrial ROS.^9^ These results have been observed in non-testicular murine tissues, and studies on OPA1 conducted in testicular tissues have been in non-human, non-murine organisms.^10^ In addition, the MICOS complex, which plays a pivotal role in dictating mitochondrial structure, is lost with age,^6,7^ thereby causing age-associated structural changes in mitochondria. However, the loss of MICOS has been little investigated in murine or human tissues, especially in human testicular tissue. To further understand the role of the complex in sperm health, we chose Mic60, as it is the central subunit of the MICOS complex and is key to mitochondrial function.^11^

Additionally, the majority of ROS production is a side effect of mitochondrial respiratory metabolism.^12^ Superoxides are the main oxygen-free radical produced in mitochondria,^13^ and if present in elevated amounts, can cause cell damage and death^14^ via oxidative damage to mitochondrial proteins and cellular components outside the mitochondria.^15^ While the relationship between mitochondrial structure and oxidative stress remains poorly understood, increased mitochondrial ROS production is age-related.^16^ Notably, many disease-related pathologies have been linked to ROS-dependent changes in mitochondria,^17^ highlighting the potential central role of mitochondria in age-related testicular and sperm pathology. Collectively, understanding these aspects of mitochondrial structure could be impactful in improving clinical applications for studying age-related defects in male reproductive health.

## METHODS

### *All of Us* Research Program Cohort Selection and Integrative Genomic Analysis

#### Study Cohort.

**Figure 1:**
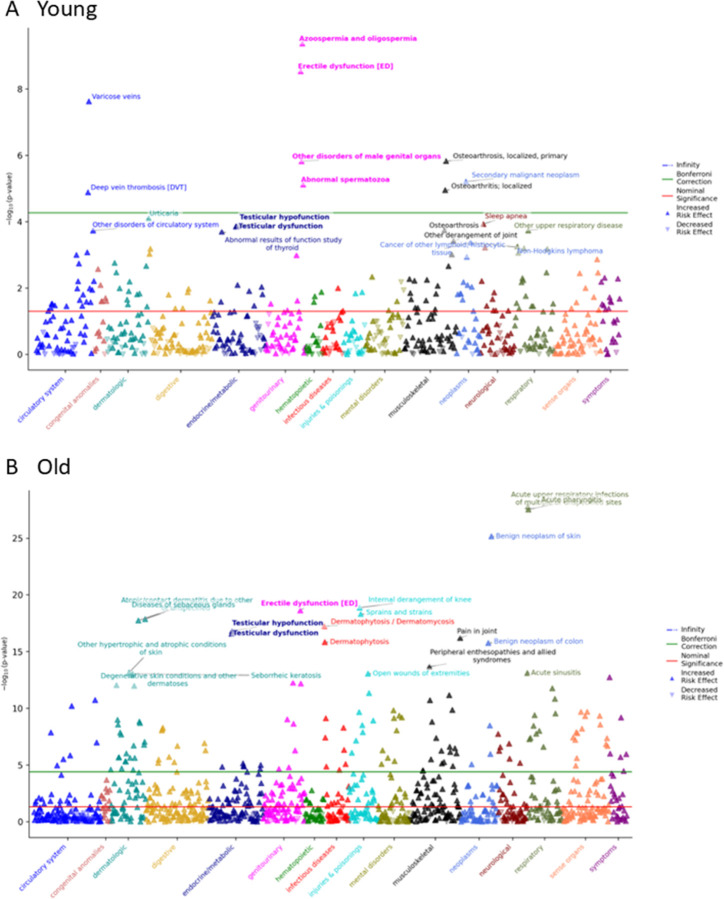
Flowchart of study cohort selection for analysis from the *All of Us* Research Program. Participants were selected from the *All of Us* Research Program using Controlled Tier Dataset version 8, including participants enrolled and consented through October 1, 2023. Among 633,248 participants aged ≥18 years at consent, exclusions were applied for age <20 years at consent, absence of linked EHR data, absence of srWGS data, and assigned sex at birth other than male or missing/declined responses. The final analytic sample comprised 121,064 participants, including 995 MI cases identified by diagnostic codes and 120,069 controls without MI. Age-stratified analyses further excluded participants aged 41–54 years to generate young (20–40 years) and old (≥55 years) case-control subsets. EHR, electronic health record; srWGS, short-read whole-genome sequencing; MI, male infertility.

Study participants were drawn from the *All of Us* Research Program, a national longitudinal cohort established by the National Institutes of Health to advance precision medicine through enrollment of a large, diverse adult population (aged ≥18 years) across the United States, initiated in 2017. All participants provided written informed consent, authorized access to their electronic health record (EHR), and completed baseline surveys that captured demographic information. Data was accessed through the *All of Us* Researcher Workbench (Controlled Tier).^18^ This study used the Curated Data Repository version 8 (CDRv8; C2024Q3R4; released February 20, 2026), which includes participants enrolled and consented through October 1, 2023. Among the 633,248 participants aged ^3^18 years or older at the time of consent, we identified 121,064 participants aged ≥ 20 years and with male sex assigned at birth at the time of consent, with available EHR data and short-read whole-genome sequencing (srWGS) data, who were eligible for inclusion in the analytic sample. Participants were excluded if they lacked EHR or srWGS data and had an assigned sex at birth other than male, or missing or declined responses. After applying inclusion and exclusion criteria, 995 and 120,069 participants were classified as male infertility (MI) cases and controls (without MI), respectively (Fig. 1). MI cases were defined by at least one occurrence of a MI diagnosis, including male infertility, failure to conceive due to infertility of male partner, or infertile male syndrome, derived from electronic health record (EHR) data using International Classification of Diseases, Ninth or Tenth Revision, Clinical Modification (ICD-9-CM/ICD-10-CM) diagnosis codes, and Systematized Nomenclature of Medicine Clinical Terms (SNOMED CT) concept sets (Table S1). Controls met the same age at consent, EHR, srWGS, and assigned sex at birth criteria, but had no MI diagnosis. For age-stratified analyses, MI cases and controls were further categorized as young (20–40 years) or old (^3^55 years). Participants aged 41–54 years were excluded from case-control subset analyses. These subsets comprised 412 young MI cases and 28,411 young controls, and 254 old MI cases and 66,638 old controls.

#### Clinical Phenome-Wide Association Studies (PheWAS).

We performed age-stratified clinical case-control phenome-wide association studies^19^ in the *All of Us* analytic cohort to identify clinical diagnoses associated with male infertility. Male infertility case status was encoded as the independent variable of interest. Analyses were conducted separately among young men aged 20–40 years and older men aged ^3^55 years; participants aged 41–54 years were excluded from age-stratified analyses. Covariates were derived using PheTK v0.1.47, an *All of Us*-adapted Python framework for phecode-based phenome-wide association testing. Models were adjusted for age at last EHR event, sex at birth, and the first ten genetic principal components. Participants with missing covariate data were excluded to generate covariate-complete analytic cohorts. ICD-9-CM and ICD-10-CM diagnosis codes from electronic health records were restricted to each age-stratified cohort and mapped to phecodes using phecode version 1.2 with U.S. ICD mapping. Logistic regression was performed for each phecode, with phecode case status modeled as the dependent variable and male infertility case status modeled as the independent variable. Phecodes with fewer than 5 cases or fewer than 2 total observations were excluded from the analysis to ensure stable model estimation. In the final age-stratified PheWAS, 925 phecodes were tested in the young cohort and 1,229 in the older cohort. Statistical significance was assessed using Bonferroni correction based on the number of phecodes tested, corresponding to thresholds of *p* < 5.41 × 10^−5^ for the young cohort and *p* < 4.07 × 10^−5^ for the older cohort. Nominal associations were defined as p < 0.05.^20^ To assess cross-stratum concordance of clinical associations, we compared phecode-specific effect estimates between the young and old PheWAS analyses using the regression beta coefficients (log-odds) from models fit separately in each age stratum. For phecodes that were nominally significant in at least one stratum (*p* < 0.05), young-stratum beta estimates were plotted on the *x*-axis and old-stratum beta estimates on the *y*-axis, with standard errors shown as error bars and a fitted line summarizing the cross-stratum relationship.

#### Clinical Lab-Wide Association Scan (LabWAS).

We performed age-stratified clinical laboratory-wide association analyses^21^ in the *All of Us* cohort to identify laboratory phenotypes associated with male infertility. Laboratory measurements were extracted from the *All of Us* measurement table and harmonized across related concepts by mapping to shared laboratory phenotype labels, assigning each to a laboratory domain, and retaining a consistent unit per phenotype. Repeated measurements were summarized at the participant level using the median value for each laboratory phenotype. Extreme values were excluded using a within-laboratory outlier filter (>4 standard deviations from the mean). Laboratory phenotypes were eligible for testing if available in at least 100 participants overall and at least 20 male infertility cases within each age stratum. Analyses were conducted separately in young (20–40 years) and older (^3^55 years) cohorts. Laboratory values were rank-based inverse-normal transformed and analyzed using linear regression, with male infertility case status as the independent variable of interest, adjusting for age at last EHR event, sex at birth, and the first 10 genetic principal components. In total, 228 laboratory phenotypes were tested. Nominal significance was defined as *p* < 0.05, and Bonferroni significance was defined as *p* < 5.05 × 10^−4^ for the young cohort and *p* < 2.75 × 10^−4^ for the older cohort. To evaluate cross-stratum concordance, we compared regression beta coefficients between age groups. Laboratory phenotypes that were nominally significant in at least one stratum were included, with young-stratum estimates plotted against old-stratum estimates and Pearson correlation used to quantify concordance.

#### Genome-Wide Association (GWAS) Analysis of Male Infertility.

We performed age-stratified genome-wide association analyses of male infertility in the *All of Us* Research Program using short-read whole-genome sequencing data from the analytic cohort. Analyses were conducted separately among young men aged 20–40 years and older men aged ^3^55 years after excluding participants aged 41–54 years from age-stratified case-control comparisons. Within each age stratum, male infertility case status was modeled as a binary outcome in relation to allele dosage using Firth logistic regression implemented in Hail v0.2 on the GRCh38 reference genome in a Spark-based cloud environment. Models were adjusted for age and the first ten genetic principal components to account for population structure. We restricted analyses to high-quality biallelic single-nucleotide polymorphisms from whole-genome sequencing data after variant-level quality control, including a genotype call rate 95% and a minor allele frequency (MAF) ^3^1%. We defined a suggestive significance threshold of *p* < 1 × 10^−5^ to prioritize loci for downstream investigation. The SNPnexus web tool was then used to identify genes associated with prioritized sites.^22^

Quantile-quantile (Q-Q) plots were used to assess model fit and genomic inflation. Q-Q plots were generated from a tail-preserving, approximately uniform random sample of association *p*-values in which all variants with *p* < 1 × 10^−5^ were retained, and all remaining variants were sampled at random with probability 5 × 10^−3^. The per-chromosome sampled tables were then unioned to construct Q-Q plots in Hail (v0.2), and genomic inflation was summarized using the genomic control factor (*λ*_*GC*_).

To compare genetic effect sizes across age strata, we matched shared variants between the young- and old-stratum GWAS summary statistics and compared per-allele beta estimates after harmonizing effect-allele orientation. Variants reaching suggestive significance in at least one stratum (*p* < 1 × 10^−5^) were highlighted, and the cross-stratum relationship was visualized by plotting young-stratum beta estimates on the *x*-axis and old-stratum beta estimates on the *y*-axis with standard errors shown as error bars and a fitted weighted regression line.

#### Mouse Testes Sample Preparation

**Figure 2:**
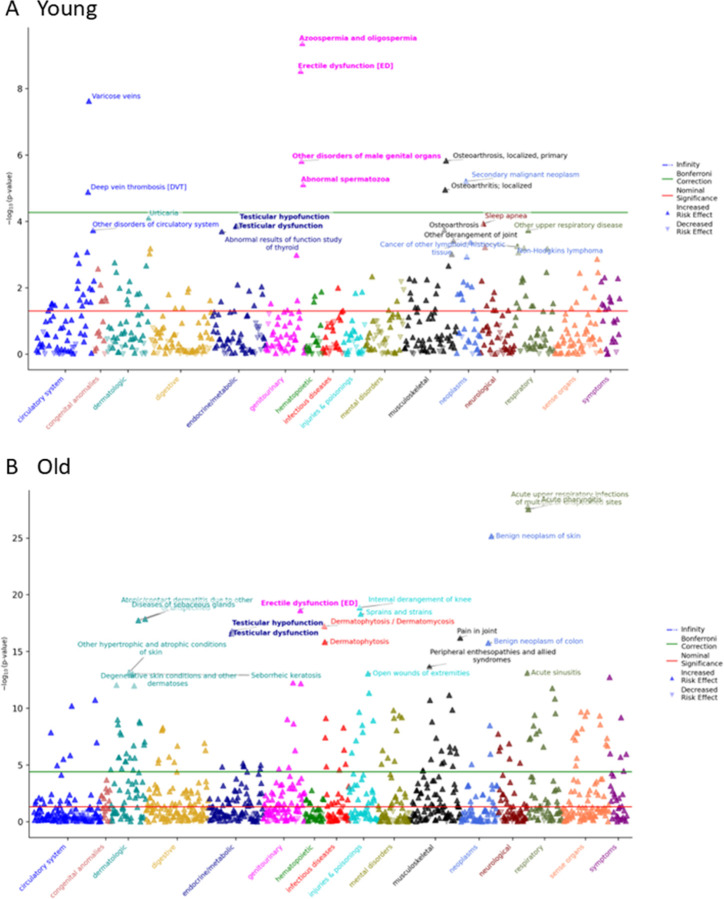
Age-stratified clinical PheWAS of male infertility in the *All of Us* Research Program. (A-B) Clinical phenome-wide association study of male infertility case status compared with non-male-infertility controls among young men aged 20–40 years (A) and older men aged ^3^55 years (B) in the *All of Us* Research Program. Each point represents a phecode tested using logistic regression, with male infertility case status modeled as the independent variable and phecode case status modeled as the dependent variable. ICD-9-CM and ICD-10-CM diagnosis codes were mapped to phecodes using phecode version 1.2. Models were adjusted for age at last EHR event, sex at birth, and the first ten genetic principal components. Phecodes are grouped by disease category on the *x*-axis, and the y-axis shows −log10(*p-*value). Upward triangles indicate phecodes associated with increased odds among male infertility cases, whereas downward triangles indicate decreased odds. Horizontal lines denote nominal significance (*p* < 0.05) and Bonferroni correction corresponding to *p* < 5.41 × 10^−5^ (young cohort) or *p* < 4.07 × 10^−5^ (older cohort). Selected Bonferroni-significant and biologically relevant male reproductive phenotypes are annotated.

Male mice were anesthetized with 5% isoflurane. Testes were removed and incubated in 2% glutaraldehyde with 100 mM phosphate buffer for 30 minutes. The testes were then dissected into 1 mm^3^ cubes and incubated in 2.5% glutaraldehyde, 1% paraformaldehyde, 120 mM sodium cacodylate solution for 1 hour. Tissues were washed three times with 100 mM cacodylate buffer at room temperature before being completely submerged in 3% potassium ferrocyanide and 2% osmium tetroxide for 1 hour at 4°C. These tissues were subsequently treated with 0.1% thiocarbohydrazide and 2% filtered osmium tetroxide for 30 minutes and were then left overnight in 1% uranyl acetate at 4°C. Between each step, 3 de-ionized water washes were performed. Samples were immersed in a 0.6% lead aspartate solution for 30 minutes at 60°C the next day, then dehydrated in graded concentrations of acetone. This dehydration was performed for 5 minutes, with each sample being successively immersed in 20%, 50%, 70%, 90%, 95%, and 100% acetone. Tissues were embedded in Epoxy Taab hard resin, embedded in fresh resin, and then polymerized at 60°C for 36–48 hours. After polymerization was complete, blocks were sectioned for TEM to identify regions of interest (ROIs), trimmed to 0.5 mm × 0.5 mm, and glued to aluminum pins; a total of 7 ROIs were selected for use across the 2 samples. Afterward, pins were placed in an FEI/Thermo Fisher Scientific Volume scope 2 SEM, a state-of-the-art SBF imaging system. With help from this SBF imaging system, 300 10 μm by 10 μm ultrathin (90 nm) serial sections were obtained from each sample using previously described techniques.^23^ All sections were collected onto formvar-coated slot grids (Pella, Redding, CA, USA), stained, and imaged as discussed in prior literature.^6,7,23–25^ SBF-SEM was used to image the mitochondria in 3D for 3-month (young) and 2-year (elderly) mice (n = 3–4; 300 mitochondria per cell type) (Figure 2). These ages were chosen for the murine samples as they have been found to correspond to 20-year-old and 70-year-old human adults, respectively.^26^

#### Manual Segmentation of Mitochondria

The software Amira (Waltham, MA, USA) was used to perform 3D reconstruction of mitochondria for visualization and quantification of mitochondrial structure in the testes at different stages of spermatogenesis. Mitochondria were manually segmented by hand with a Wacom Pen on a Wacom Tablet (Kazo, Japan), and each mitochondrion was traced from when it first appeared on the orthoslices until it either disappeared from the orthoslices or separated into a new mitochondrion or mitochondria. Mitochondria were traced from orthoslice 30 onward to avoid incomplete segmentation. Mitochondria of a total of 3 cell types (residual, germ cell, and loose sperm) were manually segmented.

#### Structure Quantifications

After manual segmentation was completed, pictures of the 3D-reconstructed mitochondria were taken of all the traced mitochondria in a given ROI as well as pictures of individual mitochondria for use in mitotyping. Videos through the x-axis and y-axis were also taken of the traced mitochondria in each ROI. Manual segmentation of mitochondria enabled Amira to generate Excel spreadsheets containing the area, perimeter, volume, sphericity, and complexity index of traced mitochondria for quantitative analysis. Sphericity (calculated as π^1/3^(6 × volume)^2/3^/(surface area)) was used to quantitatively compare mitochondria to a perfect sphere. In addition, mitochondrial complexity index was utilized to measure the complexity of the mitochondrial shape via surface area and branching relative to volume: (surface area^3^/16π^2^volume^2^).^27^ Then, mitotyping was performed. Mitotyping is a qualitative analytical technique derived from karyotyping, which involves laying out the 23 sets of human chromosomes next to each other to better understand their overall size, shape, volume, and complexity.^27^ The same was done with the traced mitochondria, with 8–10 mitochondria per ROI being randomly selected to accomplish this.

#### Mouse Sample Preparation for TEM Analysis

Cells were prepared as previously described.^24^ Samples were fixed in 2.5% glutaraldehyde in sodium cacodylate buffer for 1 hour at 37 °C, embedded in 2% agarose, postfixed in buffered 1% osmium tetroxide, stained with 2% uranyl acetate, and dehydrated through a graded ethanol series. After embedding in EMbed-812 resin, 80-nm sections were cut on an ultramicrotome and stained again with uranyl acetate as well as lead citrate.

#### Human Sample Collection and Preparation

Sperm collection was conducted in accordance with approved Brazilian IRB protocols. Additional specimens were collected and utilized under the Vanderbilt University Medical Center IRB protocols. On-the-shelf specimens from the neuropathology archive, originally obtained during routine clinical care for diagnostic purposes and not fully exhausted during testing, were also included in this study. On-the-shelf specimens in the neuropathology archive that were collected for testing at the time of biopsy but were not exhausted for diagnosis were collected during routine clinical care.

#### Drosophila Testes Dissection Protocol

Imaging was performed using a JEOL JEM-1230 transmission electron microscope at 120 kV. Mitochondria were manually traced and quantified using the freehand tool in NIH ImageJ software.^28,29^ Measurements were performed using the Multi-Measure ROI tool in ImageJ.^3,6,7,25,29^

#### Human Sample MitoTracker Analysis

100 μL of sperm was pipetted onto a 35 mm confocal dish along with 1.5 mL 1X phenol-free Dulbecco’s Modified Eagle Medium (DMEM) containing F-12 nutrient mixture. A 1:5,000 solution of MitoTracker Deep Red in medium was made, and 500 μL of the solution was added to stain sperm mitochondria. The plates were incubated at 37°C for 20 minutes before transferring to a spinning disc confocal microscope (SDCM) for fluorescent and widefield live imaging to obtain Nikon Dimension 2 files. Baseline images of different regions containing sperm were collected, and then the potent Mic60 inhibitor MicIxin was added at a final concentration of 5 μM. The cells were incubated with the inhibitor for 20 minutes before secondary imaging. Images were analyzed in the ImageJ plugin TrackMate to compare sperm motility and sperm tail morphological changes. Tail diameter was given by the “Feret” measurement, and all other measurements were standard within ImageJ.

#### ROS Analysis

ROS assays were attained from Cayman Chemical Company, and a human sperm sample was assayed in 96-well plates as explained in the company protocols. The kits were as follows: ROS Detection Cell-Based Assay Kit (DCFDA) [Cat: 601520]; Mitochondrial ROS Detection Assay Kit [Cat: 701600]; and ROS Detection Cell-Based Assay Kit (DHE) [Cat: 601290]. 20 μL of the human sperm sample was pipetted into the wells in triplicate for each assay. Wells marked for inhibitory treatment were treated with 1 μL of the respective inhibitor prior to the positive control incubation step. Optical densities for each sample were measured using a microplate reader and used as data points for constructing the graphs.

#### Drosophila Testes Preparation and Confocal Imaging

Testes were dissected from young (3-day old) and aged (65-day old) male *Drosophila melanogaster* flies expressing mitochondrial green fluorescent protein (mito-GFP) under the DJ694 GAL4 driver. The testes were fixed in a 4% paraformaldehyde in phosphate buffered saline (PBS) solution, washed, and permeabilized with Triton X 100 in PBS. Nuclei were stained with DAPI, and mito-GFP fluorescence was used to assess mitochondrial structure and distribution within the testes. The tissues were then mounted using antifade mounting medium for confocal microscopy. The images were taken using the same settings between both young and old fly testes for direct comparison of mitochondrial morphology and organization. The mitochondrial length was quantified using ImageJ.

### Statistical Analyses

Graphs were made in GraphPad Prism 972 software (La Jolla, CA, USA), with each bar representing the standard error of mean. A minimum threshold of p < 0.05 was used to represent a significant difference for all statistical analyses. Higher degrees of statistical significance (i.e., **, ***, and ****) were defined as p < 0.01, p < 0.001, and p < 0.0001, respectively. For TEM, MitoTracker, and mito-GFP analyses, comparisons were made between young and old males using the Wilcoxon rank-sum test. For the ROS assay analysis, comparisons were made between inhibitory treatments using one-way ANOVA.

## RESULTS

### Age-Stratified Clinical PheWAS Links Male Infertility to Reproductive Phenotypes in Young Men and Systemic Comorbidity in Older Men

To define the clinical phenotypic landscape of male infertility across the adult lifespan, we first performed age-stratified clinical PheWAS in the *All of Us* Research Program. The analytic cohort included 121,064 male participants with EHR and whole-genome sequencing data, including 995 male infertility cases and 120,069 controls. Age-stratified analyses were conducted in young men aged 20–40 years (Fig. 2A) and older men aged ≥55 years (Fig. 2B). In the young cohort, the young-stratum PheWAS showed a phenotype pattern dominated by male reproductive and related vascular or musculoskeletal diagnoses. Male infertility was most significantly associated with reproductive phenotypes, including azoospermia and oligospermia (OR = 395.3, *p* = 4.27 × 10^−10^), erectile dysfunction (OR = 7.29, *p* = 2.94 × 10^−9^), other disorders of male genital organs (OR = 5.16, *p* = 1.52 × 10^−6^), and abnormal spermatozoa (OR = 28.33, *p* = 7.62 × 10^−6^). Additional Bonferroni-significant associations included varicose veins, localized osteoarthrosis, secondary malignant neoplasm, and deep vein thrombosis. In contrast, the older cohort showed a broader clinical comorbidity profile. The most significant associations included acute upper respiratory infections of multiple or unspecified sites (OR = 5.65, *p* = 1.58 × 10^−28^), acute pharyngitis (OR = 8.07, *p* = 3.00 × 10^−28^), benign neoplasm of skin (OR = 5.67, *p* = 7.00 × 10^−26^), internal derangement of knee (OR = 6.32, *p* = 1.36 × 10^−19^), and erectile dysfunction (OR = 4.07, *p* = 2.39 × 10^−19^). Older men with male infertility also showed significant enrichment for dermatologic, infectious, musculoskeletal, respiratory, endocrine/metabolic, and genitourinary phenotypes, including testicular hypofunction, testicular dysfunction, prostatitis, and other disorders of male genital organs (Table S2).

Cross-stratum comparison of PheWAS effect estimates showed limited concordance between young and old male infertility strata (*r* = −0.10, *p* = 0.156; Fig. S5A). Although similar reproductive phenotype domains were represented across analyses, associations were generally more pronounced in the old stratum, whereas corresponding phenotypes in the young stratum were largely nominal. This pattern suggests age-dependent heterogeneity in the phenotypic correlates of male infertility, with potential additional contributions from differences in phenotype prevalence or statistical power. These findings suggest that male infertility presents differently across age strata. In younger men, it is mostly linked to direct reproductive phenotypes, whereas in older men it is embedded within a broader systemic comorbidity burden consistent with aging-related multisystem disease.

### Age-Stratified Clinical LabWAS Reveals Metabolic and Hematologic Differences Across the Lifespan

**Figure 3:**
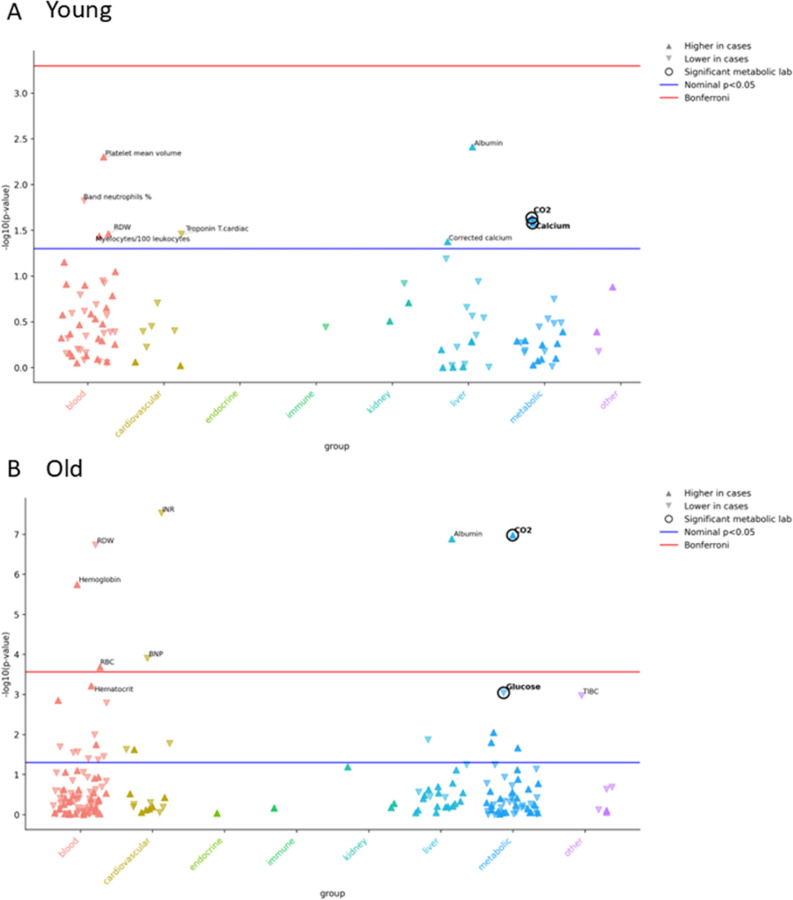
Age-stratified clinical LabWAS of male infertility in the *All of Us* Research Program. (A) Young cohort (20–40 years). (B) Older cohort (^3^55 years). Each point represents a harmonized laboratory phenotype tested using covariate-adjusted linear regression with male infertility (MI) case status as the independent variable. Laboratory values were summarized at the participant level and rank-based inverse normal transformed prior to analysis. Laboratory phenotypes are grouped by domain along the *x-*axis, and the *y*-axis represents −log10(*p*-value). Upward triangles indicate higher laboratory values in MI cases, and downward triangles indicate lower values. The blue horizontal line denotes nominal significance (*p* < 0.05), and the red horizontal line indicates the Bonferroni-corrected significance threshold for each age stratum corresponding to as *p* < 5.05 × 10^−4^ for the young cohort and *p* < 2.75 × 10^−4^ for the older cohort. Selected nominally and Bonferroni-significant laboratory phenotypes are annotated.

We next performed age-stratified clinical LabWAS to characterize laboratory phenotypes associated with male infertility (MI) across the adult lifespan and to determine whether MI is associated with systemic laboratory alterations consistent with mitochondrial, inflammatory, and metabolic dysfunction (Fig. 3). In the young cohort, laboratory associations were modest and largely nominal. The most prominent signals were observed in hematologic and inflammatory-related phenotypes, including platelet mean volume (*β* = 0.107, *p* = 1.09 × 10^−3^), band neutrophils (*β* = 0.098, *p* = 2.10 × 10^−3^), red cell distribution width (*β* = 0.075, *p* = 4.41 × 10^−3^), and myelocytes (*β* = 0.079, *p* = 1.14 × 10^−2^). Additional nominal associations included cardiac biomarkers such as troponin (*β* = 0.112, *p* = 1.06 × 10^−2^) and metabolic measures including calcium (*β* = 0.068, *p* = 1.41 × 10^−2^), carbon dioxide (*β* = −0.076, *p* = 1.60 × 10^−2^), and albumin (*β* = −0.060, *p* = 4.15 × 10^−2^). None of these associations exceeded the Bonferroni threshold, suggesting that early manifestations of MI are characterized by subtle inflammatory, hematologic, and metabolic perturbations rather than overt systemic dysfunction.

In contrast, the older cohort demonstrated a broader and more pronounced laboratory phenotype profile. Bonferroni-significant associations were observed for multiple hematologic traits, including hemoglobin (*β* = 0.185, *p* = 4.33 × 10^−6^), hematocrit (*β* = 0.183, *p* = 7.94 × 10^−6^), red blood cell count (*β* = 0.176, *p* = 1.05 × 10^−5^), and red cell distribution width (*β* = 0.136, *p* = 1.38 × 10^−5^), consistent with altered erythropoiesis. Additional significant associations included coagulation-related markers such as international normalized ratio (INR; *β* = −0.169, *p* = 3.92 × 10^−4^) and cardiometabolic biomarkers including B-type natriuretic peptide (*β* = 0.150, *p* = 5.23 × 10^−4^). Metabolic phenotypes were also enriched among older MI cases, including glucose (*β* = 0.090, *p* = 1.56 × 10^−3^), low-density lipoprotein cholesterol (*β* = 0.073, *p* = 4.01 × 10^−3^), and total cholesterol (*β* = 0.071, *p* = 5.05 × 10^−3^). Across both age strata, selected metabolic traits, including carbon dioxide and albumin, showed consistent directional effects, although effect sizes were generally larger in the older cohort (Table S3).

Cross-stratum comparison of LabWAS effect estimates showed weak concordance between young and old male infertility strata (*r* = 0.12, *p* = 0.443; Fig. S5B). Laboratory associations were more pronounced in the old stratum, which included Bonferroni-significant findings, whereas the young stratum showed no Bonferroni-significant laboratory associations. These findings suggest that laboratory correlates of male infertility are more robustly expressed in older men and are not uniformly shared across age groups. They support an age-dependent shift from relatively subtle inflammatory and hematologic changes in younger men to broader metabolic, cardiovascular, and organ-function abnormalities in older men. This pattern is consistent with the possibility that mitochondrial- and oxidative-stress-related mechanisms contribute to age-dependent differences in male infertility and its systemic correlates.

### Age-Stratified GWAS Identifies Distinct Genetic Signals for Male Infertility Across Young and Older Men

We next performed age-stratified GWAS of male infertility to determine whether the age-dependent clinical heterogeneity observed in the PheWAS and LabWAS was accompanied by differences in genetic association patterns (Fig. 5). No variants reached conventional genome-wide significance in either age stratum. In the young-stratum analysis, nominal target-enriched signals localized near genes with known roles in mitochondrial structure, cristae organization, and reproductive biology, including loci mapping to *OPA1*, *CHCHD6*, and *MIC60/IMMT*, supporting a younger-onset pattern enriched for mitochondrial and spermatogenic biology. In contrast, the older-stratum GWAS showed a broader suggestive signal distribution, consistent with the more systemic clinical phenotype profile observed in older men. In the older stratum, the full GWAS included 7,253,978 analyzed variants, with a minimum *p* value of 8.42 × 10^−7^. Sixty-seven variants met the suggestive threshold of *p* < 1 × 10^−5^. When the older-stratum results were restricted to the curated target-gene list, 1,617 nominal target-matching rows were retained, but none of these target-matching variants reached the suggestive threshold (Table S4). Comparison of young- and old-stratum GWAS effect estimates showed limited concordance, supporting age-dependent heterogeneity in the genetic architecture of male infertility (Fig. S6). These findings suggest that younger-onset male infertility may be more enriched for mitochondrial and reproductive biology, whereas older-onset male infertility reflects broader and more heterogeneous genetic contributions.

### Clinical Measurements Demonstrate Reduced Semen Quality and Fertility in Older Men

To first determine whether aging alters standard clinical semen parameters, we assessed pH, ejaculate volume, liquefaction time, and macroscopic appearance across young and aged cohorts (Fig. 5A–D, H–K). Despite clear differences in donor age distribution, these bulk semen characteristics remained largely unchanged. Semen pH was comparable between groups, and no significant differences were observed in ejaculate volume or liquefaction time. Similarly, macroscopic features were consistent across cohorts, with most samples classified as grayish white and opalescent in color and appearance, respectively (Fig. 5B). We also used reported TEM images (Fig. 5E–F) to measure mitochondrial area (Fig. 5G) between young and old patients. There was a strong decrease in area in elderly patients, suggesting a correlation between aging and fusion-fission dynamics. Together, these findings indicate that aging does not substantially disrupt overall seminal fluid composition or accessory gland function but does affect factors regulating mitochondrial size.

To determine the functional consequences of aging on sperm behavior, we next assessed sperm motility across defined motility classes from this cohort data. Quantitative analysis demonstrated a significant increase in the proportion of immotile sperm as indicated by Motility Grade 0 (Fig. 5M) in aged individuals, accompanied by a marked reduction in properly coordinated movement as indicated by Motility Grades III & IV (Fig. 5P). Consistent with these findings, the overall sperm motility index (Fig. 5Q) was significantly decreased in aged samples, indicating a global decline in motility performance. We also examined whether aging impacts sperm production and developmental output. We observed a reduction in spermatozoa, Kruger strict morphology, sperm concentration, rapid progressive concentration, and sperm count in aged samples compared to young ones (Fig. 5S–W). Together, these data demonstrate that aging shifts sperm populations toward reduced progressive movement and impaired developmental processes, highlighting functional deficits that is not captured by bulk semen parameters alone.

### MicIxin Inhibition Drastically Impairs Motility and Shift ROS Production of Human Sperm

To further determine how sperm motility is affected, we stained clinical samples with MitoTracker Deep Red and live imaged cells with SDCM before and after treatment with MicIxin to mimic the effects of age-related structural deterioration. Representative imaging revealed clear differences in movement dynamics between untreated and treated samples (Fig. 6A–B) whereby untreated sperm exhibited greater speed and directionality (Fig. 6C–E), but efficacy in the ability to coordinate this movement was not significantly impacted (Fig. 6F–G), suggesting that aging selectively impacts specific motility states rather than uniformly impairing all movement behaviors. The images also revealed that MicIxin inhibition produced major tail kinks that increased curvature (Fig. 6H–I), which could explain the effects in reduced motility. We found that the treated sperm exhibited significantly greater shifts in the angle of the tails in relation to the characteristic straight tails of normal sperm (Fig. 6J). However, tail diameter remained unaffected (Fig. 6K), suggesting a greater role for tail bending in interference with sperm motility. Surprisingly, the sperm midpieces seemed to be unaffected by MicIxin treatment (Fig. 7C–G), suggesting that the structure-related motility shift is due to the tail itself rather than the midpiece, a key component to overall flagellar activity.

Excessive ROS production is a hallmark male infertility, which may be attributed to mitochondrial dysfunction. We measured ROS production in human sperm samples to identify changes due to MICOS inhibition. Although generally not significant, we noticed marked increases in cytosolic and mitochondrial ROS production between untreated and treated samples (Fig. 7H–J). This could potentially highlight a connection between mitochondrial stress and infertility.

#### Drosophila Testes Reveal Conserved Mitochondrial Remodeling Associated with Reproductive Aging

Age-associated mitochondrial remodeling was highly conserved across species in *Drosophila melanogaster* testes and mirrored many of the structural phenotypes identified in mammalian reproductive tissues. To investigate whether aging alters mitochondrial organization in spermatids, confocal imaging was performed in MEF2 Gal4>UAS mito-GFP flies expressing mitochondrially targeted GFP in testicular tissue. Comparative analyses were conducted between young flies at 3 days of age and aged flies at 65 days of age. Testes were stained with DAPI to visualize nuclei, while endogenous mito-GFP fluorescence was used to assess mitochondrial architecture and distribution throughout spermatid structures (Fig. 8B–C).

In young Drosophila testes, spermatids displayed highly organized and elongated mitochondrial networks distributed uniformly throughout developing germ cells (Fig. 8D). Mitochondrial structures appeared filamentous, interconnected, and spatially coordinated around spermatid nuclei, suggesting preservation of mitochondrial integrity and bioenergetic organization during early adulthood. The mito-GFP signal in young flies was robust and demonstrated continuous mitochondrial patterning throughout the spermatid cytoplasm, consistent with efficient mitochondrial maintenance and proper organelle distribution required for sperm maturation and motility. Nuclear morphology within young testes also appeared highly organized with normal DAPI staining patterns and minimal structural disruption.

In contrast, aged testes from 65-day-old flies exhibited marked alterations in mitochondrial architecture and organization. Aged spermatids showed fragmented, disorganized mitochondrial structures with reduced mitochondrial network continuity compared with young controls (Fig. 8E). Mitochondrial distribution appeared uneven and disrupted throughout the spermatid cytoplasm, with evidence of clustering, shortened mitochondrial morphology, and loss of coordinated spatial organization. These aging-associated mitochondrial alterations were accompanied by reductions in the elongated filamentous mitochondrial structures observed in young testes, suggesting impaired mitochondrial maintenance and remodeling during reproductive aging. Importantly, the aged mitochondrial phenotypes identified in Drosophila testes strongly paralleled the mitochondrial remodeling patterns observed in mammalian sperm and testicular tissues throughout the study (Fig. 8F–I). These conserved cross-species findings support the concept that aging induces fundamental changes in mitochondrial architecture within reproductive tissues and further implicate mitochondrial structural dysfunction as a major contributor to age-associated reproductive decline.

### Aging Impacts Mitochondrial Architecture in Mouse Testis

SBF-SEM and 3D reconstruction were conducted to elucidate changes in parameters such as area, perimeter, volume, sphericity, and complexity index between young and old samples. Orthoslices demonstrated elaborate tissue and mitochondrial structure (Fig. S2A-B, S3A-B, S4A-B). 3D reconstruction of the mitochondria helped make the spatial distribution of mitochondria within the tissue (Fig. S2A’-B’, S3A’-B’, S4A’-B’) and in relation to one another (Fig. S2A”-B”, S3A”-B”, S4A”-B”) evident. Compared to the young control (3-month), both aged residual mitochondria of testicular tissue and aged loose sperm cells demonstrated decreases in area, perimeter, volume, and complexity index, as well as a significant increase in sphericity during spermatogenesis, all indicative of fragmentation (Fig. S2C-G, S4C-G). All these changes were significant except for the decrease in loose sperm cell volume, which had no significance. Germ cells of testicular tissue, on the other hand, demonstrated exact opposite significant changes in these same parameters: increases in area, perimeter, volume, and complexity index paired with a decrease in sphericity (Fig. S3C-G). Volume-based mitotyping revealed that in germ cell and loose sperm cell types, aged mitochondria were more fragmented than their young counterparts (Fig. S3H, S4H).

Together, these data demonstrate that aging drives a multi-layered impairment across the reproductive axis, spanning reduced spermatogenic output, decreased sperm abundance, and diminished functional and structural integrity. This coordinated decline suggests that aging impacts both sperm production and performance, rather than a single isolated parameter.

## DISCUSSION

Our study integrates ultrastructural, functional, and population-scale analyses to identify mitochondrial architectural remodeling as a central determinant of male reproductive decline. While traditional semen parameters remained largely preserved with age, we observed profound alterations in sperm motility and morphology in association with mitochondrial organization and ROS production. Together, these findings position mitochondrial structure as a potential key regulator of sperm fertility across an individual’s lifespan.

To further define the clinical signatures associated with male infertility, we performed a lab-wide association study (LabWAS) across the All of Us cohort, stratified by age. This approach enabled systematic evaluation of circulating biomarkers and clinical laboratory values associated with infertility-related phenotypes. In the younger cohort, significant associations were primarily observed within hematologic and inflammatory markers. Notably, platelet mean volume, red cell distribution width (RDW), band neutrophil percentage, and myelocyte counts were elevated in cases compared to controls. These findings suggest that early manifestations of male infertility are associated with subtle alterations in immune and hematopoietic function, consistent with low-grade systemic stress or inflammation. Additional associations with cardiac-related markers, including troponin T, further point to early systemic perturbations that extend beyond the reproductive axis.

In contrast, the older cohort exhibited a markedly different and more pronounced laboratory signature, characterized by significant alterations in metabolic and organ function markers. Among the most significant associations were increased glucose levels, altered CO_2_ concentrations, and changes in albumin, indicating disruptions in metabolic homeostasis and systemic physiology. Additional markers, including red blood cell indices, hematocrit, hemoglobin, and brain natriuretic peptide (BNP), further suggest broader involvement of cardiovascular and metabolic systems in individuals with infertility. Importantly, the magnitude and significance of these associations were greater in the older cohort, consistent with a progressive model in which early, subtle changes in inflammatory pathways evolve into widespread metabolic dysfunction with age. This shift from immune-dominated signatures in younger individuals to metabolic and organ-level dysregulation in older individuals highlights a temporal progression in the systemic manifestations of infertility.

These findings strongly align with the mitochondrial mechanisms identified in our experimental analyses. Mitochondrial dysfunction, particularly impaired oxidative phosphorylation and increased reactive oxygen species (ROS) production, is known to influence both inflammatory signaling and metabolic regulation. The enrichment of hematologic and inflammatory markers in younger individuals is consistent with early mitochondrial stress responses, whereas the metabolic abnormalities observed in older individuals reflect downstream consequences of sustained mitochondrial dysfunction. Together, the LabWAS analysis reveals that male infertility is associated with distinct, age-dependent systemic biomarker profiles. These data provide population-level evidence linking mitochondrial dysfunction to both early inflammatory changes and later-stage metabolic decline, reinforcing the concept that infertility represents a multi-system condition rooted in impaired cellular bioenergetics.

A major point of this work is the application of multiple microscopic approaches to assess reproductive health through mitochondrial architecture and sperm morphology and motility. Young samples exhibited elongated, structurally complex mitochondria, which is characteristic of an organized network optimized for energy production. By contrast, aged sperm contained smaller, more spherical mitochondria.

Quantitative analyses of these microscope images demonstrated that aging and MICOS disruption lead to reductions in mitochondrial area, perimeter, circularity index, and volume, accompanied by increased sphericity. The mouse SBF-SEM data revealed that these shifts were most pronounced in later stages of sperm development, indicating that mitochondrial dysfunction progressively worsens during maturation. Confocal microscopy further demonstrated this phenomenon in the aging *D. melanogaster* model. We observed an increased dysregulated framework in mitochondrial complexity and network organization in the older flies when compared to the young ones, indicating that the same phenotypic trends occur across multiple species. Furthermore, human sperm were found to demonstrate the same phenotypes in the clinical observations, which was accompanied by observations of major shifts in motility upon MicIxin treatment. Notably, these shifts were not associated with midpiece morphological changes despite the high concentration of mitochondria in the midpiece, suggesting a predominant role of tail structure in effective movements characteristic of male fertility. Collectively, these data demonstrate that aging induces conserved mitochondrial architectural remodeling across species. The integration of Drosophila imaging with mammalian sperm phenotyping, human genetic analyses, and ultrastructural mitochondrial reconstruction further strengthens the conclusion that mitochondrial remodeling is a central, evolutionarily conserved feature of age-associated reproductive dysfunction. The Drosophila model also provided important mechanistic and translational support for the broader hypothesis that mitochondrial and MICOS-dependent remodeling pathways contribute to reproductive aging phenotypes. Because Drosophila reproductive tissues undergo highly coordinated mitochondrial elongation, shaping, and redistribution during spermatid maturation (Fig. 8A), the observed aging-associated disruptions in mito-GFP organization suggest that aging may impair mitochondrial dynamics, cristae maintenance, organelle communication, and energy distribution within spermatids. These findings are particularly important given the high energetic demands of sperm maturation and motility, and they further support the overall model linking mitochondrial structural dysfunction to impaired reproductive fitness during aging.

This study also examined structural changes in the murine testis and sperm associated with aging using SBF-SEM and 3D reconstruction. Aged residual mitochondria in the testis and loose sperm mitochondria displayed decreases in area, perimeter, volume, and complexity index, as well as an increase in sphericity. On another note, as previously mentioned, mitochondrial fragmentation is known to occur during aging and is driven by increased fission activity. Fission has been shown to decrease mitochondrial area, perimeter, volume, and complexity index, while increasing mitochondrial sphericity.^6,7^ Thus, these results are indicative of mitochondrial fragmentation as well. Germ cells exhibited opposite changes in these parameters with age, an unexpected finding indicative of higher rates of fusion that inhibit fragmentation, resulting in dynamic mitochondria and potentially a hyperfused network. This result is uncommon but has been observed in mitochondrial studies of long-lived individuals (about 100 years old), where mitochondrial remodeling via increased mass serves to compensate for functional limitations.^12^ This result might also be due to the fact that loose sperm and residual testicular mitochondria are more similar to one another than to germ cells. This can be seen in figures depicting spermatogenesis. In this figure, germ cells correspond to 1 and 2, loose to 4, and residual to 5 (Figs. S2-S3). Thus, something could be occurring during the germ cell-to-spermatid transition that causes this shift in the trends of the physical parameters. Moreover, this unusual trend of increasing mitochondrial area, perimeter, volume, and complexity index and decreasing sphericity with age has been found in disease states such as heart failure,^6,7^ suggesting that stressed mitochondria may respond to this stress in a cell-type-dependent manner.

Mechanistically, these structural changes are highly consistent with disruption of MICOS and OPA1. They control the dynamics of cristae structure (Vue *et al*., 2023), one of the MICOS complex’s primary functions (Anand and Reichert, 2021). These proteins is essential for maintaining efficient oxidative phosphorylation, and its disruption has been linked to decreased ATP production and increased ROS generation. Because of this, loss of mitochondrial complexity and increased sphericity would be expected to impair respiratory efficiency and promote oxidative stress. We confirmed this speculation by measuring sperm ROS production after MicIxin and OPA1 inhibition, which demonstrated marked increases from the control group.

Importantly, these mitochondrial defects provide a potential mechanistic explanation for the functional impairments observed in sperm motility. ATP generated within midpiece mitochondria is required to sustain flagellar movement, and reduced bioenergetic capacity directly limits motility. Additionally, elevated ROS contributes to lipid peroxidation and protein damage that further compromise sperm quality. The combination of reduced ATP production and increased ROS predicted by MICOS disruption provides a mechanistic basis for the clinical biomarkers enriched in individuals with infertility-related phenotypes. Moreover, the age-dependent increase in infertility prevalence is corroborated by our 3DEM findings, which demonstrate progressive accumulation of mitochondrial defects across the spermatogenic axis. Importantly, the integration of genomic data suggests that these processes are not limited to aging alone. Variants in MICOS components and related mitochondrial regulators may predispose younger individuals to similar structural and functional defects, recapitulating aging-associated phenotypes in the absence of chronological aging. This shines light on mitochondrial architecture as a critical point of convergence between genetic susceptibility and age-related decline.

While this study produced robust findings, one notable limitation is that cristae structure was not examined. This is important because aging has been shown to alter cristae morphology,^27^ and changes in cristae structure might affect the degree of change observed in the mitochondrial physical parameters used in this study. Changes in cristae morphology with age should be measured using these same parameters, as well as the cristae score, a measurement assigned to cristae based on the percentage of cristae deemed normal in the sample.^27^ Moreover, OPA1 and the MICOS complex control the dynamics of cristae structure,^6,7^ with this being one of the MICOS complex’s primary functions.^30^ Accordingly, comparing the effects of knockout of these genes on the physical size parameters of cristae with their wildtype counterparts in aged testicular and sperm cells would help elucidate the extent to which age-related loss of OPA1 and MICOS affects cristae structure, rather than other age-related changes.

Future directions include elucidating the mechanisms underlying age-related changes in testicular tissue and sperm. Metabolomics can measure metabolic changes associated with aging and inactivity of MICOS subunits. Lipidomics could identify changes in levels of specific lipid classes and lipid chain lengths with age, which would affect membrane permeability, fluidity, and functionality in aged samples.^6,7^ Age-related metabolic and lipid alterations are expected to ultimately mirror those resulting from the loss of MICOS complex subunits. Lastly, transcriptional and translational expression should be assessed in these samples to identify genes associated with age-dependent mitochondrial changes.

Ultimately, more research is needed on the structure and function of testicular tissue and sperm at the organelle level. For instance, a recent study found that the centriole region of the sperm is near the sperm’s midpiece. Learning the location of the centriole in sperm is important because it is crucial for embryo development and is introduced into the embryo solely via the sperm.^31^ As the centriole plays a role in cell division and microtubule organization, it also promotes cilia and flagella assembly, thereby aiding sperm motility. We do not know how Ca^2+^ affects the sperm centriole,^32^ which is crucial to discover given that Ca^2+^ ions are also known to impact sperm motility. However, the effect of calcium depends on the stage of sperm maturation.^33^ This highlights the importance of different stages of spermatogenesis as they are structurally and functionally different. Further research in this area could guide therapeutic measures to maintain or increase mitochondrial volume and function, thereby improving male reproductive health.

## Supplementary Files

This is a list of supplementary files associated with this preprint. Click to download.

**Figure 4:**
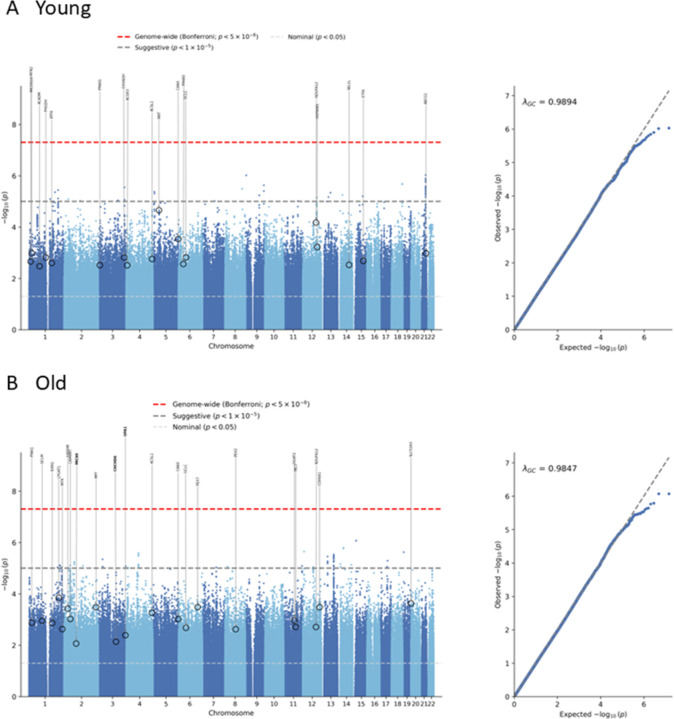
Age-stratified genome-wide association (GWAS) analyses of male infertility in the *All of Us* Research Program. (A) Young-stratum GWAS among men aged 20–40 years. (B) Old-stratum GWAS among men aged ^3^55 years. For each age stratum, left panels show Manhattan plots of genome-wide association results from Firth logistic regression of male infertility case status on allele dosage, adjusted for age and the first ten genetic principal components. Right panels show corresponding quantile-quantile (Q-Q) plots with genomic inflation factor (*λGC*). Variants were restricted to quality-controlled biallelic SNPs from whole-genome sequencing data. The dashed horizontal line marks conventional genome-wide significance (*p* = 5 × 10^−8^), and the lower dashed line marks suggestive significance (*p* = 1 × 10^−5^). Selected biologically relevant loci are annotated, including mitochondrial/cristae-related candidate genes from the nominal analysis. Genomic coordinates are based on GRCh38.

**Figure 5:**
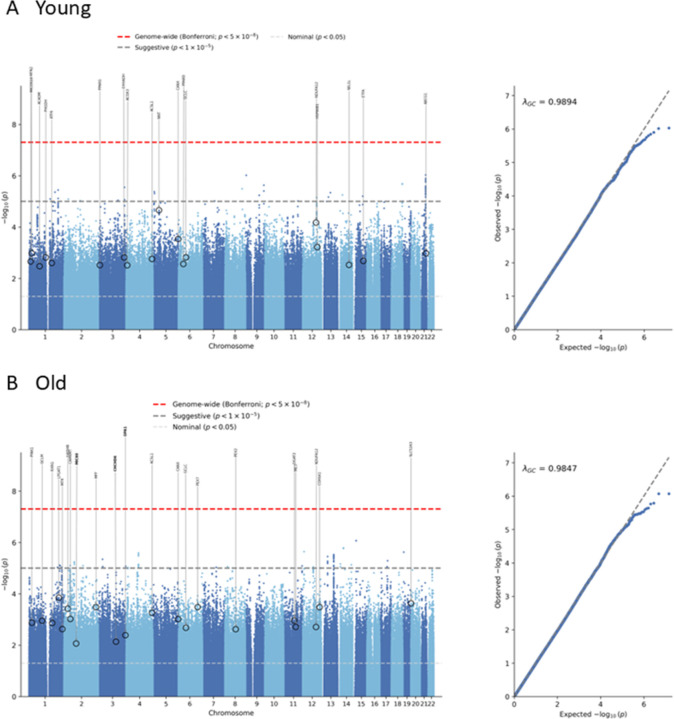
The impact of aging on sperm characteristics. (A) Semen samples were collected from young (n=28) and old (n=22) patients under routine clinical care. (B) Macroscopic appearance between young and old groups. (C-D): Widefield images of (C) young and (D) old sperm. (E-F): x10k magnification TEM images of (E) young and (F) old sperm heads and midpieces. (G) Quantification of mitochondrial area from TEM images. (H-K): Clinical metrics of patient (H) age, (I) semen liquefaction time, (J) semen volume, and (K) semen pH. (L) Illustration of motile vs. immotile sperm contributing to male infertility. (M-Q): Clinical measurements of sperm motility grades and collective sperm motility index. (R) Illustration of spermatogenesis. (S-W): Clinical measurements of semen to indicate health in sperm production through (S) number of spermatogenesis cells per cubic millimeter, (T) % of sperm following Kruger strict morphology, (U) concentration, (V) rapid progressive concentration, and (W) total sperm count.

**Figure 6:**
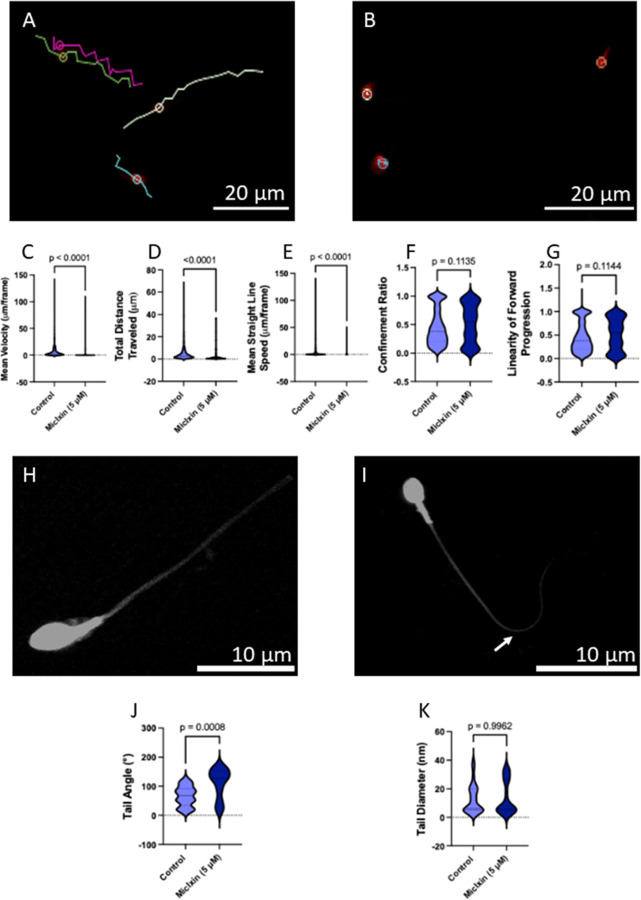
The impact of MICOS inhibition on sperm movement dynamics and tail structure. (A-B): Confocal imaging tracking sperm movement between (A) control and (B) MicIxin-treated cells. (C-G): TrackMate measurements of motility and movement efficacy before and after MicIxin treatment through (C) mean velocity, (D) total distance traveled, (E) mean straight line speed, (F) confinement ratio, and (G) linearity of forward progression. (H-I): Confocal images depicting (H) control and (I) MicIxin-treated sperm cells, emphasizing tail orientation. (J) Angle of sperm tail from midpiece to tail end. (K) Diameter of tail between midpiece and tail end.

**Figure 7:**
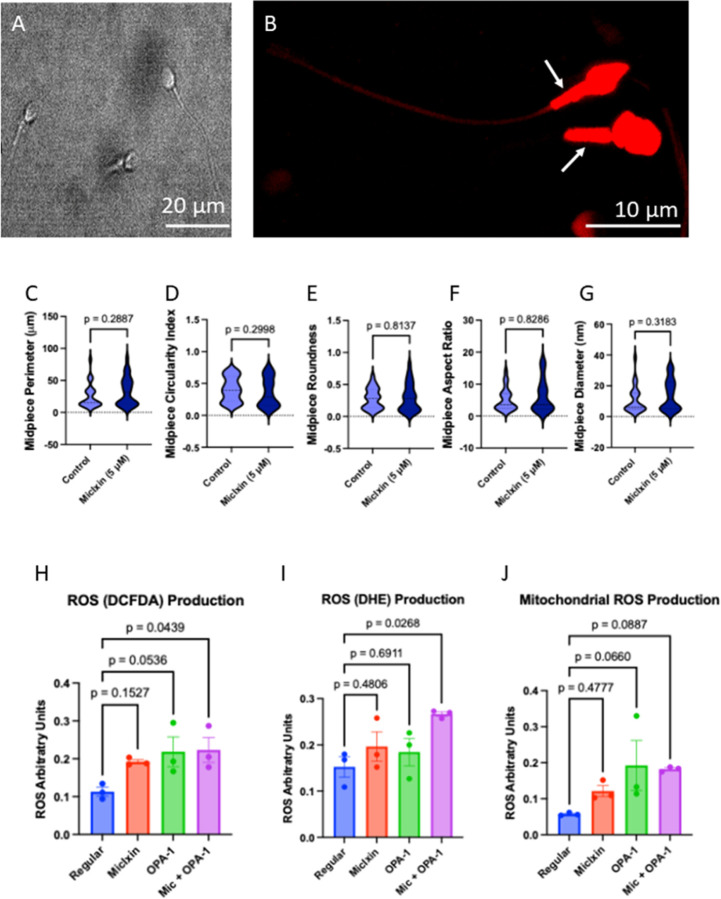
The impact of MICOS disruption on midpiece structure and oxidative stress in sperm cells. (A) Widefield image of MitoTracker-stained sperm. (B) MitoTracker image depicting intense fluorescence at midpiece. (C-G): ImageJ measurements of midpiece structure for (C) perimeter, (D) circularity index, (E) roundness, (F) aspect ratio, and (G) diameter. (H-J): Comparisons of ROS produce before and after inhibitory treatment with MicIxin and OPA-1.

**Figure 8:**
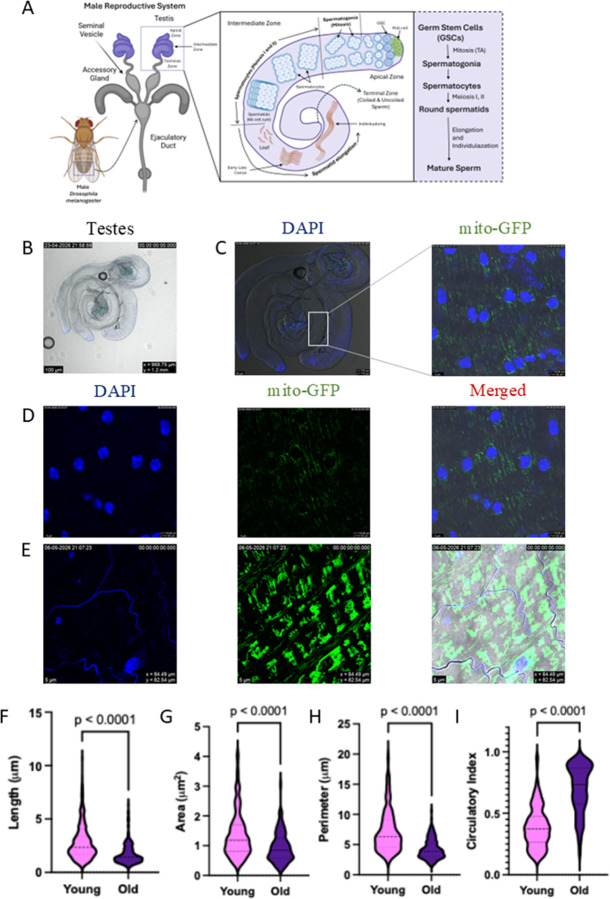
Age-associated changes in spermatid mitochondrial architecture of *Drosophila melanogaster*. (A) Schematic illustrating the steps of *Drosophila melanogaster* spermatogenesis. (B-C): Confocal images of dissected testis cross-sections in (B) widefield and (C) fluorescent microscopy with an inset of its mitochondria and nuclei. (D-E): Confocal images of spermatids stained with DAPI for nuclei and endogenously expressed mito-GFP for mitochondria from (D) 3-day-old and (E) 65-day-old *mef2-Gal4>UAS-mito-GFP* flies. (F-I): Comparisons of mitochondrial (F) length, (G) area, (H) perimeter, and (I) circularity index between 3-day-old (F: n=355; G-I: 166) and 65-day-old (F: n=266; G-I: 194) flies. Scale bar: 5μm

- FigS1.tif
- FigS1.tif
- FigS3.tif
- FigS4.tif
- FigS4.tif
- FigS6.tif
- TableS1.tif
- Supplement.docx

## Data Availability

The data that support the findings of this study are available from the corresponding author, AHJ, upon reasonable request. To protect participant privacy, *All of Us* data used in this study are available only to registered researchers through the *All of Us* Researcher Workbench, which can be accessed via https://workbench.researchallofus.org/login, subject to institutional agreements (e.g., a Data Use and Registration Agreement), required training, and data use policy compliance. Analysis code can be shared with authorized *All of Us* Researcher Workbench users upon reasonable request.
